# Solubility affects IL-1β-producing activity of the synthetic candidalysin peptide

**DOI:** 10.1371/journal.pone.0273663

**Published:** 2022-08-30

**Authors:** Taiki Mori, Hideo Kataoka, Gen Tanabe, Takeshi Into

**Affiliations:** Department of Oral Microbiology, Division of Oral Infection Health Sciences, Asahi University School of Dentistry, Mizuho, Gifu, Japan; Louisiana State University, UNITED STATES

## Abstract

Candidalysin, a peptide toxin produced specifically from hyphae of *Candida albicans*, plays a crucial role in *C*. *albicans* pathogenesis in the oral cavity and vagina. Synthetic peptides have been widely used in previous studies to investigate the bioactivity of candidalysin. Although the solubility of the peptide, which is expected to have a hydrophobic property, has not been well characterized, candidalysin solutions are usually prepared in water. In this study, we prepared the synthetic peptide candidalysin in water (CLw) or in dimethyl sulfoxide (CLd) and compared their cytotoxicity and interleukin (IL)-1β-producing activity to determine whether the activity of the peptide would be affected. In addition, we evaluated whether the NOD-like receptor family pyrin domain-containing 3 (NLRP3) inflammasome pathway or other pathways were involved in their activities. Unexpectedly, we found that CLw was not completely solubilized and contained abundant insoluble microparticles. CLw was active at comparably high concentrations (≥ 10 μM). In contrast, CLd is completely solubilized and sufficiently active at low concentrations, that is, 1 μM or less. CLw showed weak cytotoxicity and NLRP3-dependent and cathepsin B-dependent IL-1β-producing activity, whereas CLd showed strong cytotoxicity and cathepsin B-dependent IL-1β-producing activity. Fractionation of CLw revealed that NLRP3-dependent activity was caused by insoluble microparticles. Furthermore, nanoparticle tracking of CLd revealed that the peptide was present as nanoparticles with a size of 96 nm. CLw contained a small amount of such nanoparticles. Thus, the bioactivities of the synthetic peptide candidalysin, especially the IL-1β-producing activity, are affected by the solubility of the peptide depending on the solvent employed. The NLRP3-dependent activity of the synthetic peptide is caused by insoluble microparticles and may not be the intrinsic activity of candidalysin.

## Introduction

A fungous peptide toxin called candidalysin is produced specifically from the hyphae of *Candida albicans*, an opportunistic pathogen in humans, which can assist the invasion of *C*. *albicans* in the tissue of the oral cavity and vagina [1]. This peptide is also called Ece1-III because it is produced as a third of the eight cleaved fragments of cell elongation 1 (Ece1) protein that is specifically expressed in the hypha [2]. The candidate peptide is supposedly amphipathic and consists of 31 amino acids containing hydrophobic amino acid assemblies on two α-helical structures [2, 3]. To date, the bioactivity of candidalysin has been investigated from a variety of aspects, especially using an Ece1-deficient strain of *C*. *albicans* and the synthetic peptide.

The amphipathic property of candidalysin likely facilitates the pore-forming activity of this toxin that causes cell membrane damage and necrotic cell lysis in macrophages and epithelial cells [2, 4, 5]. Candidalysin-induced cell death occurs through rapid production of reactive oxygen species (ROS), disruption of mitochondrial membrane potential, ATP depletion, and release of cytochrome *c*, but not through the apoptotic machinery such as activation of caspase-8 and caspase-3 [6]. Nonetheless, candidalysin-induced cell death should be further elucidated.

In addition to its cytotoxic properties, candidalysin can induce the production of interleukin (IL)-1β through sensor protein NOD-like receptor family pyrin domain-containing 3 (NLRP3)-dependent inflammasome activation [7]. Generally, NLRP3 inflammasome-mediated IL-1β production requires two independent intracellular signaling events [8, 9]. First, a priming signal is activated through the recognition of a microbial component, such as fungal β-glucan and bacterial lipopolysaccharide (LPS), by an innate immune receptor, which drives nuclear factor (NF)-*κ*B-dependent transcription of pro-IL-1β and NLRP3. Subsequently, a triggering signal can be activated after NLRP3 recognition of a variety of exogenous and endogenous molecules, such as extracellular ATP, pore-forming toxins, viruses, bacterial cells, and the bacterial toxin nigericin. NLRP3 activation also involves cellular damage responses, including mitochondrial dysfunction, ROS generation, ion flux (Ca^2+^ influx and K^+^/Cl^−^ efflux), and lysosomal damage [9]. These events lead to NLRP3 oligomerization and assembly of an inflammasome protein complex consisting of the apoptosis associated speck-like (ASC) adaptor protein and the precursor of the cysteine protease caspase-1. The inflammasome complex serves as a platform for the activation of caspase-1, which in turn promotes the processing of pro-IL-1β for extracellular release of a mature form of IL-1β. The NLRP3-dependent triggering signal is also activated through cellular damage by particulate or crystalline matter, such as monosodium urate crystals, alum, and asbestos [10]. The mechanism by which candidalysin can stimulate NLRP3 has not been sufficiently investigated.

Although the solubility of the synthetic candidalysin, which is expected to have hydrophobic properties, has not been adequately investigated, solutions have been usually prepared in water. In this study, we investigated the solubility of the peptide in water and in an amphiphilic organic solvent, dimethyl sulfoxide (DMSO). Additionally, we compared their biological activities, particularly the cytotoxicity and IL-1β-producing activity, to determine whether their activity would be affected. In addition, we evaluated whether the NLRP3 inflammasome pathway or other pathways were involved in the activity of the peptide in different solvents. Thus, this work provides novel insights that can help to properly assess the bioactivity of the synthetic candidalysin peptide.

## Materials and methods

### Preparation of peptide solutions

The synthetic candidalysin (SIIGIIMGILGNIPQVIQIIMSIVKAFKGNK) was obtained from Peptide Institute. The peptide was dissolved in deionized water or cell culture grade DMSO (Sigma-Aldrich). The amount of water or DMSO needed to dissolve 1 mg of candidalysin to make a 1 mM solution is 302 μl. The Ece1-II peptide (DVAPAAPAAPADQAPTVPAPQEFNTAITK), a second fragment of the Ece1 protein, was synthesized by Hokkaido System Science and used as a control peptide. Ece1-II was dissolved in deionized water. The amount of water needed to dissolve 1 mg of Ece1-II to make a 1 mM solution is 350 μl. Fractionation of 100 μl of the peptide stock solutions was performed by centrifugation at 100 × *g* for 5 min at room temperature. The upper fraction (80 μl) and the residual lower fraction (20 μl) were separated by discreet pipetting. The upper fraction was undiluted, and the lower fraction was diluted five-fold to restore it to the original volume and used equally with the uncentrifuged peptide solution.

### Predicted tertiary structures and hydropathy

The predicted tertiary structure of the peptide was determined using PEP-FOLD3 (https://bioserv.rpbs.univ-paris-diderot.fr/services/PEP-FOLD3/). The grand average of hydropathicity (GRAVY) index value and hydropathy score were calculated using Expasy ProtParam (https://web.expasy.org/protparam/) and ProtScale (https://web.expasy.org/protscale/), respectively, with a window size of three amino acids.

### Detection of particles in peptide solutions

To observe microparticles in the peptide solutions, a BX41 microscope equipped with a DP21 camera (Olympus) was used. Images were obtained at 200 × magnification. The particle number/area (mm^2^) was determined using the particle analyzer tool in the ImageJ software (version 1.51, http://rsb.info.nih.gov/ij/index). The microparticles in the peptide solutions were also investigated by forward and side scatter analyses using an EC800 cell analyzer (Sony Biotechnology). A liquid-borne microparticle counter KL-05 (Rion; measurable particle size: 1.3–100 μm) was used to count the number and measure the diameter of the microparticles in the peptide solution. To analyze the nanoparticles in the peptide solution, a NanoSight NS300 nanoparticle characterizer (Malvern Panalytical; measurable particle size: 10–1,000 nm) was used according to the manufacturer’s instructions.

### Reagents

LPS from *Escherichia coli* O111:B4 was purchased from Sigma–Aldrich. The cathepsin B inhibitor Ca074Me was obtained from the Peptide Institute. Nigericin, ionomycin, and the NLRP3 inhibitor, MCC950, were obtained from AdipoGen Life Sciences. The caspase-1 inhibitor Z-YVAD-fmk and pan-caspase inhibitor Z-VAD-fmk were purchased from Abcam. The actin polymerization inhibitor cytochalasin D was obtained from Cayman Chemical and was used as a phagocytosis inhibitor. All inhibitors were dissolved in DMSO.

### Cells

The human monocytic cell line THP-1, obtained from RIKEN BioResource Research Center, was cultured in RPMI 1640 medium supplemented with 10% fetal bovine serum, penicillin G (100 units/ml) and streptomycin (100 μg/ml) in a humidified atmosphere containing 5% CO_2_ at 37°C, as described previously [11]. The cells were differentiated into macrophage-like attached cells by incubation with 20 nM phorbol 12-myristate 13-acetate (Sigma) for 24 h. After a medium change, the cells were further incubated for 24 h and used for treatment with the peptide solution in a serum-free medium. For experiments using inhibitors, cells were incubated with each compound for 1 h prior to treatment with the peptide solution.

### Cytotoxicity assay

Cytotoxicity was assessed colorimetrically using lactate dehydrogenase (LDH) release assays. Differentiated THP-1 cells were seeded in 96-well plates (2×10^4^ cells/well) and treated with peptide solution in serum-free RPMI medium. After incubation, the culture supernatants were collected to quantify LDH release by measuring the absorbance at 490 nm using the Cytotoxicity LDH Assay Kit-WST (Dojindo). The absorbance at maximum LDH release was obtained using a complete cell lysate. The absorbance of the control LDH release was obtained from the cells incubated with the vehicle (1 or 2% DMSO). Blank absorbance was determined from serum-free RPMI medium without cells. After subtracting the blank absorbance, the percentage of cytotoxicity was calculated as  100 × (experimental LDH release–control LDH release) / (maximum LDH release–control LDH release).

Cell membrane damage was assessed by fluorescent dye imaging. Cells were seeded in 24-well plates (1×10^5^ cells/well) and treated with the peptide solution. Calcein-AM and propidium iodide (PI; Dojindo) were added to the culture to stain viable and damaged cells, respectively. Hoechst33342 (Molecular Probes) was also added to stain all cells. Fluorescent images were obtained as described previously [12]. The number of PI-positive cells/area (mm^2^) was determined using the ImageJ software.

### Measurement of IL-1β

Differentiated THP-1 cells seeded in 96-well plates (2×10^4^ cells/well) were primed with 100 ng/mL LPS for 4 h before the medium was changed to serum-free RPMI 1640 medium. After treatment of cells with the peptide solution or nigericin, the culture supernatants were collected to measure IL-1β concentrations using an IL-1β Human Uncoated enzyme-linked immunosorbent assay (ELISA) Kit (Thermo Fisher Scientific).

### Western blotting

Differentiated THP-1 cells seeded in 12-well plates (1.0 × 10^6^ cells/well) were primed with 100 ng/mL LPS for 4 h, followed by treatment with the peptide solution or nigericin for 3 h. Culture supernatants were collected and concentrated using AMICON Ultra-10 filters (Millipore). After removal of the supernatant, the cells were lysed with radioimmunoprecipitation assay (RIPA) buffer (Sigma-Aldrich). The specimens were separated by sodium dodecyl sulfate-polyacrylamide gel electrophoresis (SDS-PAGE) and transferred to PVDF membranes. Immunoblotting was essentially performed as described previously [13]. The membranes were incubated with primary antibodies specific for IL-1β (D3U3E, Cell Signaling Technology), cleaved IL-1β (D3A3Z, Cell Signaling Technology), or ɑ/β-tubulin (Cell Signaling Technology). After washing, membranes were incubated with horseradish peroxidase-conjugated secondary antibodies. Immunoreactive bands were visualized using the ECL Advance western blotting substrate (Thermo Fisher Scientific) and a Light-Capture II imaging analyzer (ATTO).

### Statistical analysis

Data are expressed as means ± standard deviation (SD) and were analyzed using one-way analysis of variance (ANOVA) followed by Dunnett’s multiple comparison test.

## Results

### Solubility of the synthetic candidalysin peptide

**Fig 1:**
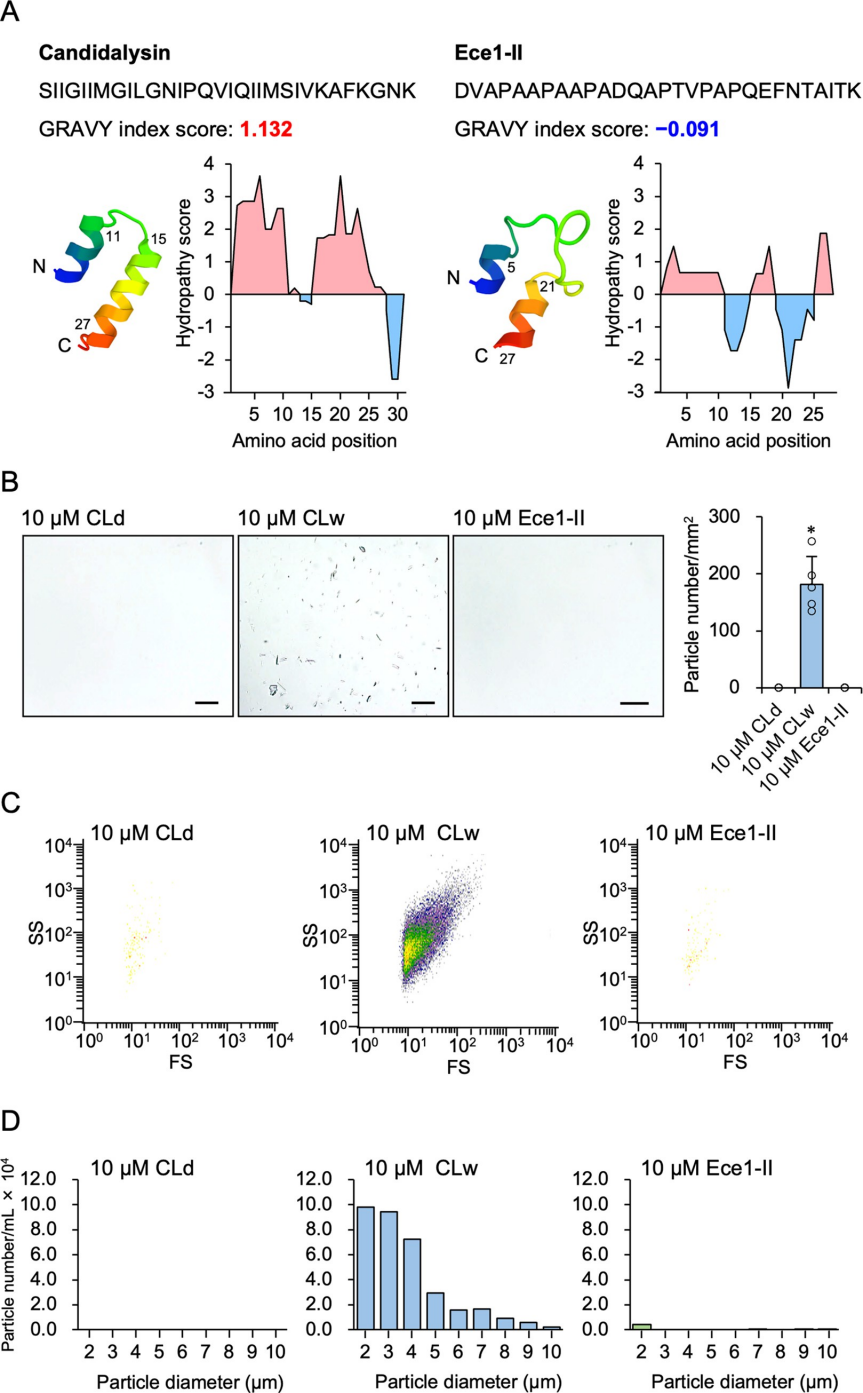
Solubility of the synthetic candidalysin peptide. A: Amino acid sequence, putative tertiary structure, GRAVY index score, and hydropathy plot with Kyte-Doolittle scale of candidalysin (left) and Ece1-II (right). A positive hydropathy score reflects an enrichment of hydrophobic amino acids. B: Microscopic images of 10 μM CLd, CLw, and the Ece1-II control peptide dissolved in water. Images were taken at 200× magnification. Scale bar, 100 μm. The quantified data of particle number/mm^2^ in the peptide solutions is shown in the chart. Data are presented as the mean ± SD (n = 5) and are representative of three independent experiments. *P < 0.05 compared with 10 μM Ece1-II by one-way ANOVA followed by Dunnett’s test (μc < μi). C: Plots of the particles detected in 10 μM CLd, CLw, and Ece1-II by the flowcytometric forward scatter (FS) and side scatter (SS) analyses. D: The number and size of microparticles in 10 μM CLd, CLw, and Ece1-II quantified using a microparticle counter. Data are representative of three independent experiments.

In most previous studies, the synthetic candidalysin was dissolved in water and used in a variety of experiments at a concentration of 10 μM or higher [2, 7, 14, 15]. Candidalysin is an amphipathic peptide with two highly hydrophobic α-helices [2]. Our analyses revealed the hydrophobic property of candidalysin with a high GRAVY index and hydropathy scores, in contrast to the hydrophilic property of the control peptide Ece1-II (Fig 1A).

To test the solubility of the synthetic candidalysin, we prepared stock solutions of the peptide dissolved in water (CLw) and others dissolved in DMSO (CLd) at concentrations of 200 μM ~ 1 mM and diluted it with water. Visually, CLw seemed substantially cloudy and contained abundant suspended particulate matter, even when diluted to a concentration of 10 μM or less. The particulate matter was difficult to dissolve despite vigorous mixing or heating. In contrast, CLd appeared transparent and completely solubilized at concentrations ≥200 μM, even when diluted with water. The control peptide, Ece1-II, was soluble in water. Microscopically, a large amount of particulate matter was observed in 10 μM CLw, whereas CLd and Ece1-II at the same concentrations did not have any particles (Fig 1B). The particles of CLw could be found at a concentration of at least 0.1 μM, and they were insoluble even when DMSO was added at concentrations of ≤10%. Additionally, flow cytometric forward and side scatter analyses revealed that CLw particles have various sizes and shapes (Fig 1C). Furthermore, microparticles with sizes ranging from to 2–10 μm were detected in 10 μM CLw using a microparticle counter. For example, 29,583 particles/mL of 5 μm particles, and 192,333 particles/mL of 3 μm particles were detected (Fig 1D). Thus, a considerably large amount of insoluble microparticles was present in CLw, suggesting the possibility that such a property affects the known bioactivity of candidalysin.

### Cytotoxicity of CLd and CLw

**Fig 2:**
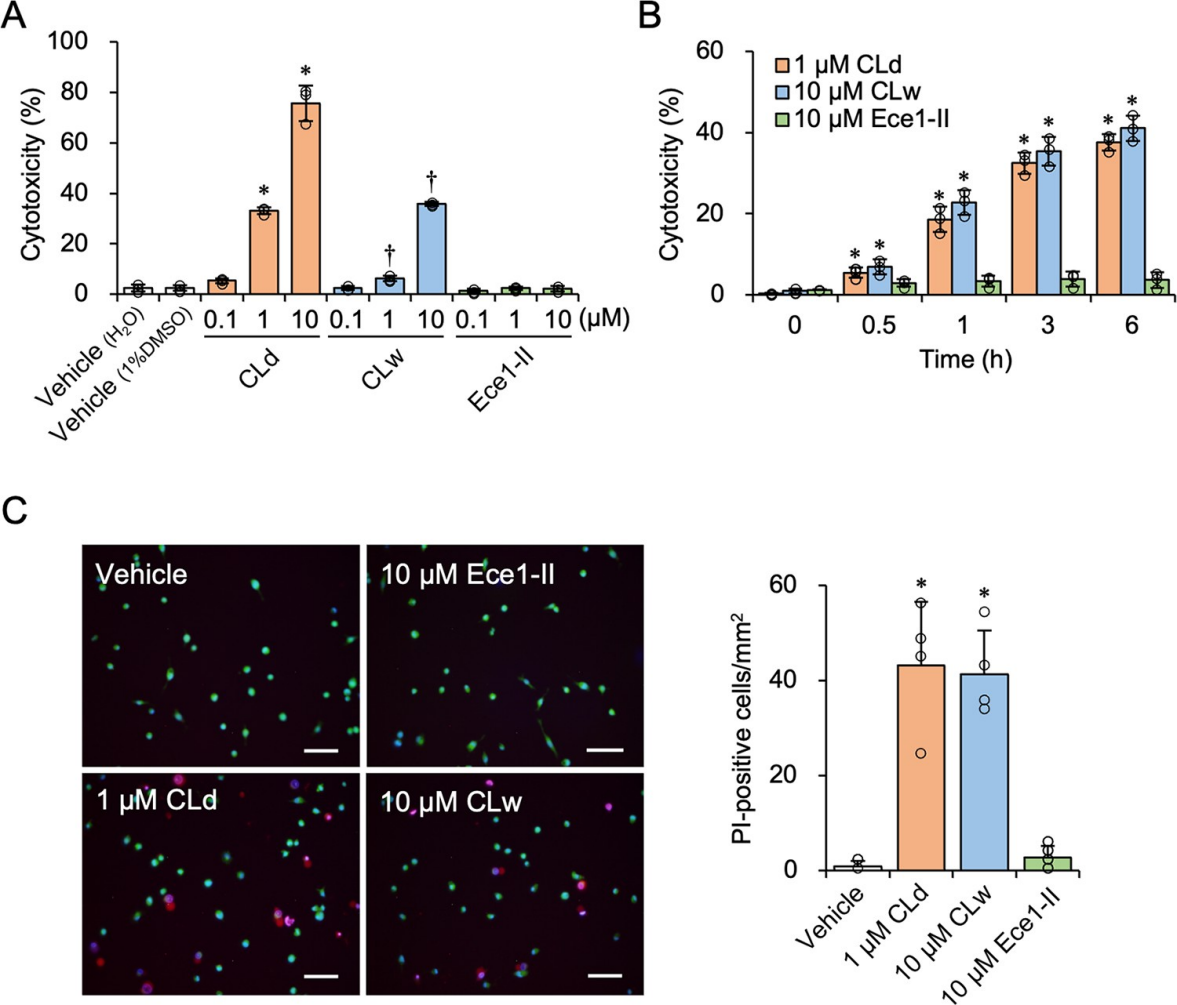
Cytotoxicity of CLd and CLw in differentiated THP-1 cells. A: Cells were treated with varied concentrations of CLd, CLw, or Ece1-II for 3 h. The vehicle controls for CLd and CLw are a medium containing 1% DMSO and a water-added medium (H_2_O), respectively. Cytotoxicity was quantified by a LDH release assay. Data are presented as the mean ± SD (n = 3) and are representative of three independent experiments. *P < 0.05 compared with the vehicle (1% DMSO; μc < μi); ^†^*P* < 0.05 compared with the vehicle (H_2_O; μc < μi) by one-way ANOVA followed by Dunnett’s test. B: Cells were treated with 1 μM CLd, 10 μM CLw, or 10 μM Ece1-II for the indicated period (0−6 h). Cytotoxicity was quantified by an LDH release assay. Data are presented as the mean ± SD (n = 3) and are representative of three independent experiments. *P < 0.05 compared with 0 h by one-way ANOVA followed by Dunnett’s test (μc < μi). C: Cells were treated with 1 μM CLd, 10 μM CLw, or 10 μM Ece1-II for 3 h, followed by staining with PI (red, indicative of damaged cells), Calcein-AM (green, indicative of live cells), and Hoechst 33342 (blue, all cells). The vehicle control is a medium containing 1% DMSO. Fluorescence images were taken with a fluorescence microscope at 100× magnification. Scale bars, 100 μm. Quantified data (the number/mm^2^ of PI-positive cells) are presented as the mean ± SD (n = 4) and are representative of three independent experiments. *P < 0.05 compared with the vehicle by one-way ANOVA followed by Dunnett’s test (μc < μi).

We compared the bioactivities of CLd and CLw. As candidalysin has cytotoxic properties [2, 7], we examined the cytotoxicity using an LDH release assay in differentiated THP-1 macrophage-like cells. CLd and CLw showed dose-dependent cytotoxicity, but Ece1-II did not exhibit cytotoxicity (Fig 2A). The activity of 10 μM CLd was stronger than that of 10 μM CLw, which showed similar activity to 1 μM CLd. The cytotoxicity of CLd and CLw increased gradually after stimulation and peaked at approximately 6 h (Fig 2B). Monitoring of intracellular calcium for a 600 s period revealed that gradual calcium influx can be induced by 10 μM CLd, but not by 1 μM CLd and 10 μM CLw (S1 Fig), suggesting that the cytotoxic activities of CLd and CLw were somewhat slow. PI staining revealed that CLd and CLw exerted cell membrane damage, but their activities were clearly observed approximately 3-h after stimulation (Fig 2C). Thus, CLd and CLw share similar cytotoxic properties, but the activity of CLd is approximately 10-times stronger than that of CLw.

**Fig 3:**
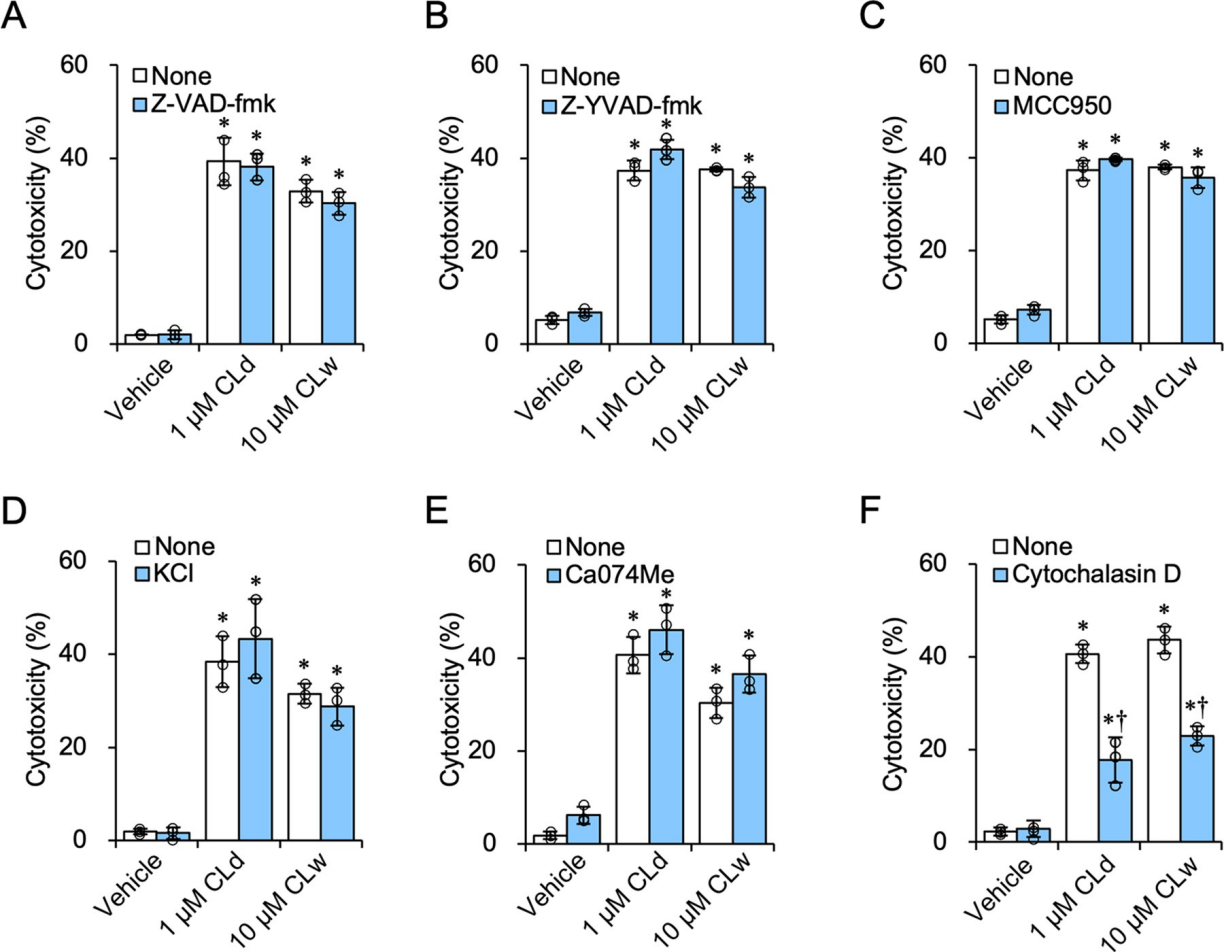
Influence of the inhibitors on the cytotoxicity of CLd and CLw. The differentiated THP-1 cells were pretreated with the following inhibitors: 20 μM of the pan-caspase inhibitor Z-VAD-fmk (A); 20 μM of the caspase-1 inhibitor Z-YVAD-fmk (B); 2 μM of the NLRP3 inhibitor MCC950 (C); 25 mM KCl for inhibition of K^+^ efflux (D); 20 μM of the cathepsin B inhibitor Ca074Me (E); and 20 μM of the phagocytosis inhibitor cytochalasin D (F) or with vehicle for 1 h. The vehicle control is a medium containing 2% DMSO. Cells were then treated with 1 μM CLd or 10 μM CLw for 3 h. Cytotoxicity was quantified by LDH release assay. Data are presented as the mean ± SD (n = 3) and are representative of three independent experiments. *P < 0.05 compared with the vehicle (μc < μi); ^†^*P* < 0.05 compared with the none (μc > μi) by one-way ANOVA followed by Dunnett’s test.

To further determine the different cell-death-inducing properties of CLd and CLw, several types of inhibitors were used. Inhibition of caspases by the pan-caspase inhibitor Z-VAD-fmk, which can suppress apoptotic and necroptotic cell death, did not affect the cytotoxicity of CLd and CLw (Fig 3A). Furthermore, cytotoxicity was not influenced by the caspase-1 inhibitor Z-YVAD-fmk (Fig 3B), the NLRP3 inhibitor MCC950 (Fig 3C), inhibition of potassium efflux by 25 mM potassium chloride treatment (Fig 3D), and the cathepsin B inhibitor Ca074Me (Fig 3E), all of which are related to the NLRP3 inflammasome pathway. Meanwhile, the inhibition of phagocytosis by cytochalasin D reduced the cytotoxicity of CLd and CLw (Fig 3F). This is partly consistent with a previous result showing that cytochalasin D suppresses the cytotoxicity of the candidalysin peptide dissolved in water [7]. These results indicated that there was no obvious difference in the cell death-inducing properties of CLd and CLw in THP-1 cells.

### IL-1β-producing activity of CLd and CLw

**Fig 4:**
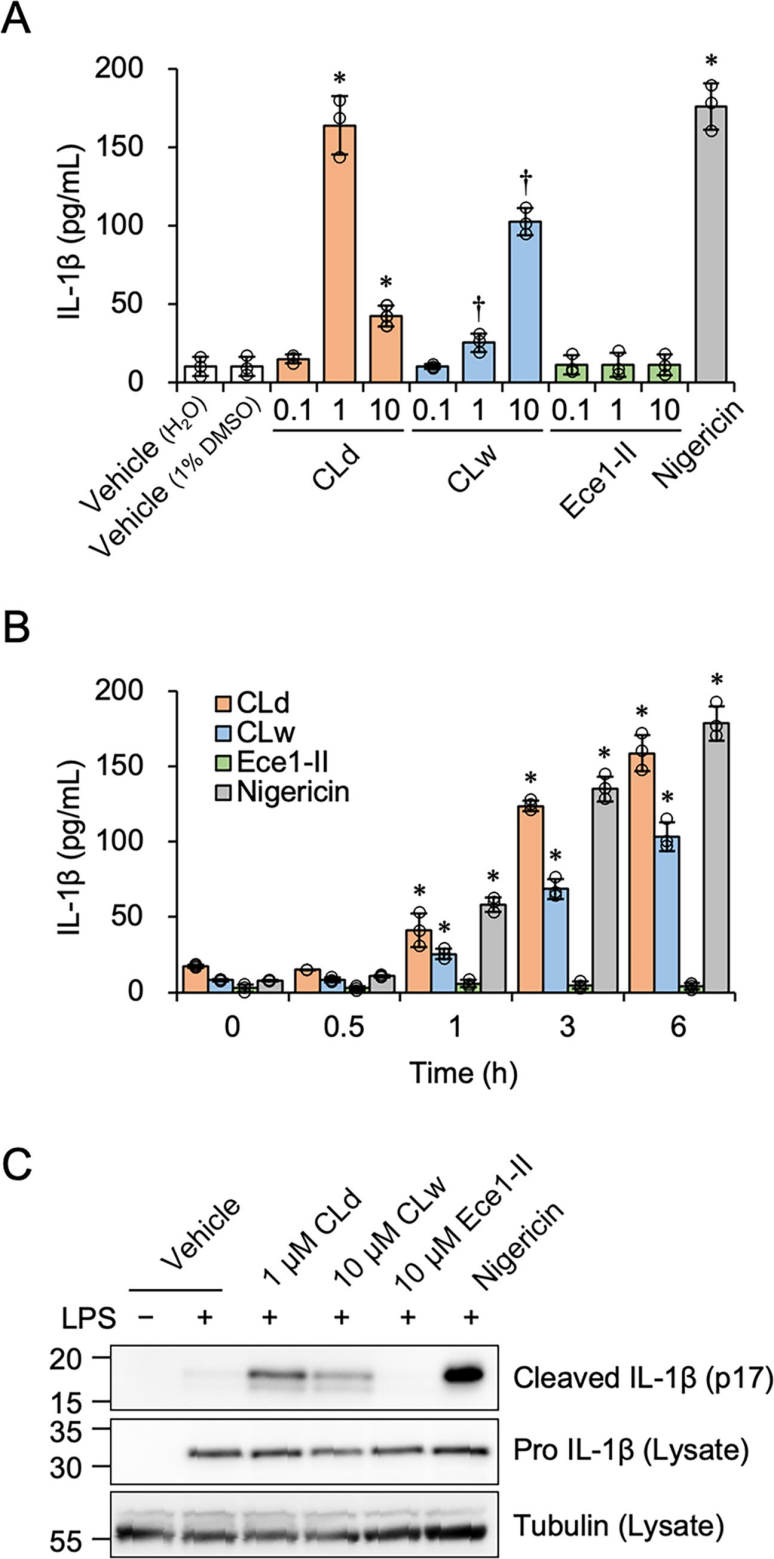
The IL-1β-producing activity of CLd and CLw in LPS-primed THP-1 macrophage-like cells. A: Cells were treated with varied concentrations of CLd, CLw, Ece1-II, or 1 μM nigericin for 3 h. The vehicle controls for CLd and CLw are a medium containing 1% DMSO and a water-added medium (H_2_O), respectively. The IL-1β levels in the culture supernatants were assessed by ELISA. Data are presented as the mean ± SD (n = 3) and are representative of three independent experiments. *P < 0.05 compared with the vehicle (1% DMSO; μc < μi); ^†^*P* < 0.05 compared with the vehicle (H_2_O; μc < μi) by one-way ANOVA followed by Dunnett’s test. B: Cells were treated with 1 μM CLd, 10 μM CLw, 10 μM Ece1-II, or 1 μM nigericin for the indicated period (0−6 h). The IL-1β levels in the culture supernatants were assessed by ELISA. Data are presented as the mean ± SD (n = 3) and are representative of three independent experiments. *P < 0.05 compared with 0 h by one-way ANOVA followed by Dunnett’s test (μc < μi). C: Cells were treated with 1 μM CLd, 10 μM CLw, 10 μM Ece1-II, or 1 μM nigericin for 3 h. The vehicle control is a medium containing 1% DMSO. Cells that were not primed with LPS were also prepared. Culture supernatants were collected for the detection of the mature form of 17-kDa IL-1β (cleaved IL-1β; p17), and cell lysates were used for the detection of pro-IL-1β and tubulin by immunoblot analyses. Representative results of at least three independent experiments are shown. The uncropped images are shown in S1 Raw images.

Candidalysin is able to stimulate the NLRP3 inflammasome to activate caspase-1, facilitating the extracellular release of IL-1β by cleaving pro-IL-1β into the mature 17-kDa form [16, 17]. Next, we investigated the activities of CLd and CLw in inducing extracellular IL-1β production. Although the cells were primed with LPS to stimulate intracellular pro-IL-1β induction, this process did not affect the cytotoxicity of CLd and CLw (S2 Fig). Extracellular IL-1β production was strongly induced by 1 μM CLd, the level of which was similar to that induced by the positive control nigericin (Fig 4A). The level of IL-1β production was lowered by 10 μM CLd (Fig 4A), probably due to its potent cytotoxicity (Fig 2A). CLw induced dose-dependent IL-1β production, but its activity was weaker than that of 1 μM CLd. IL-1β induction by 1 μM CLd and 10 μM CLw gradually increased over 6 h (Fig 4B). Immunoblot analyses revealed that CLd and CLw were able to induce cleavage of intracellular pro-IL-1β into the mature 17-kDa form, and the activity of 1 μM CLd was stronger than that of 10 μM CLw (Fig 4C).

**Fig 5:**
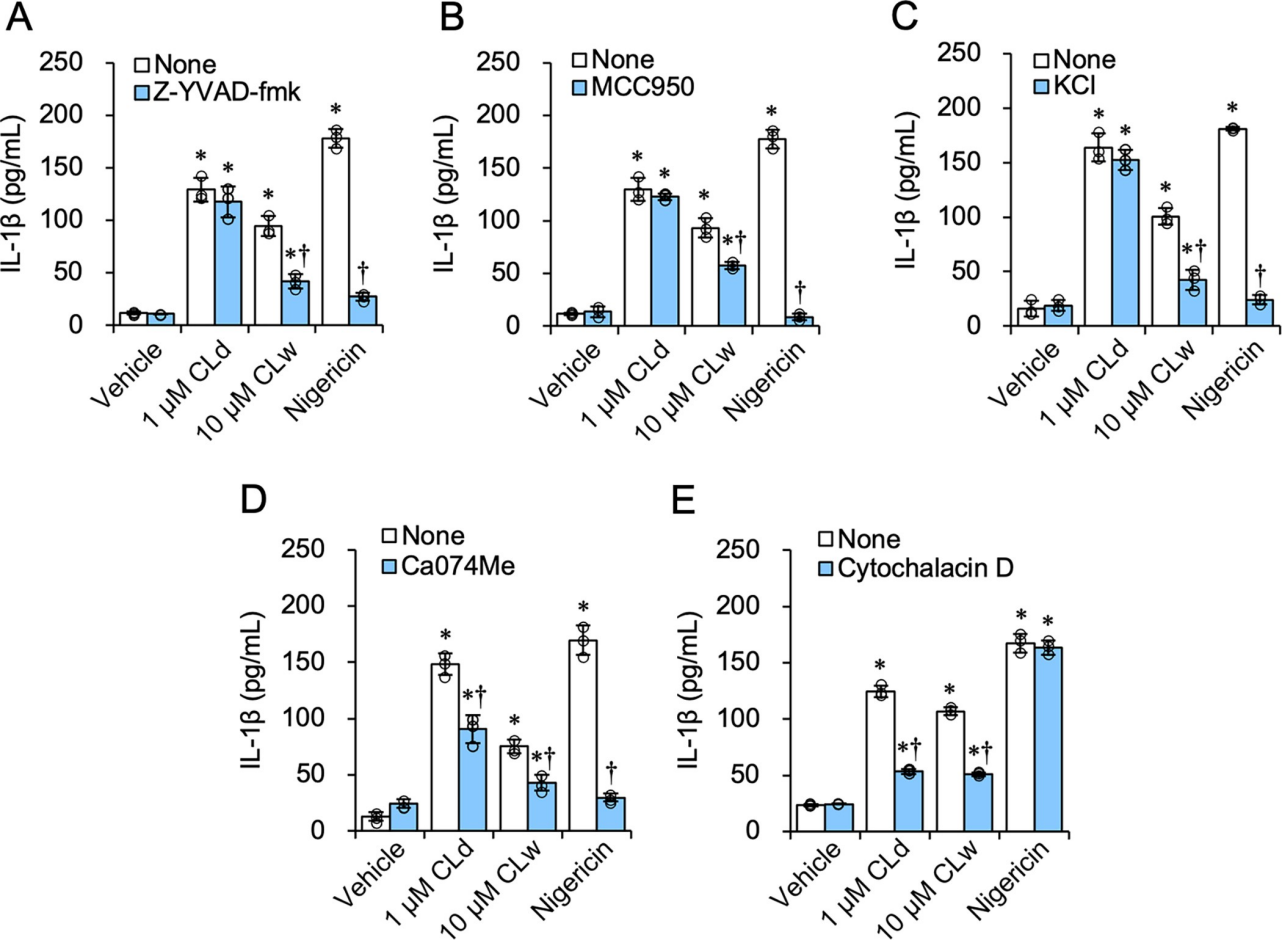
Influence of the inhibitors on the IL-1β-producing activity of CLd and CLw. LPS-primed THP-1 cells were pretreated with following inhibitors: 20 μM of the caspase-1 inhibitor Z-YVAD-fmk (A); 2 μM of the NLRP3 inhibitor MCC950 (B); 25 mM KCl for inhibition of K^+^ efflux (C); 20 μM of the cathepsin B inhibitor Ca074Me (D); and 20 μM of the phagocytosis inhibitor cytochalasin D (E) or with vehicle for 1 h. The vehicle control is a medium containing 2% DMSO. Cells were then treated with 1 μM CLd or 10 μM CLw for 3 h. The IL-1β levels in the culture supernatants were assessed by ELISA. Data are presented as the mean ± SD (n = 3) and are representative of three independent experiments. *P < 0.05 compared with the vehicle (μc < μi); ^†^*P* < 0.05 compared with the none (μc > μi) by one-way ANOVA followed by Dunnett’s test.

**Fig 6:**
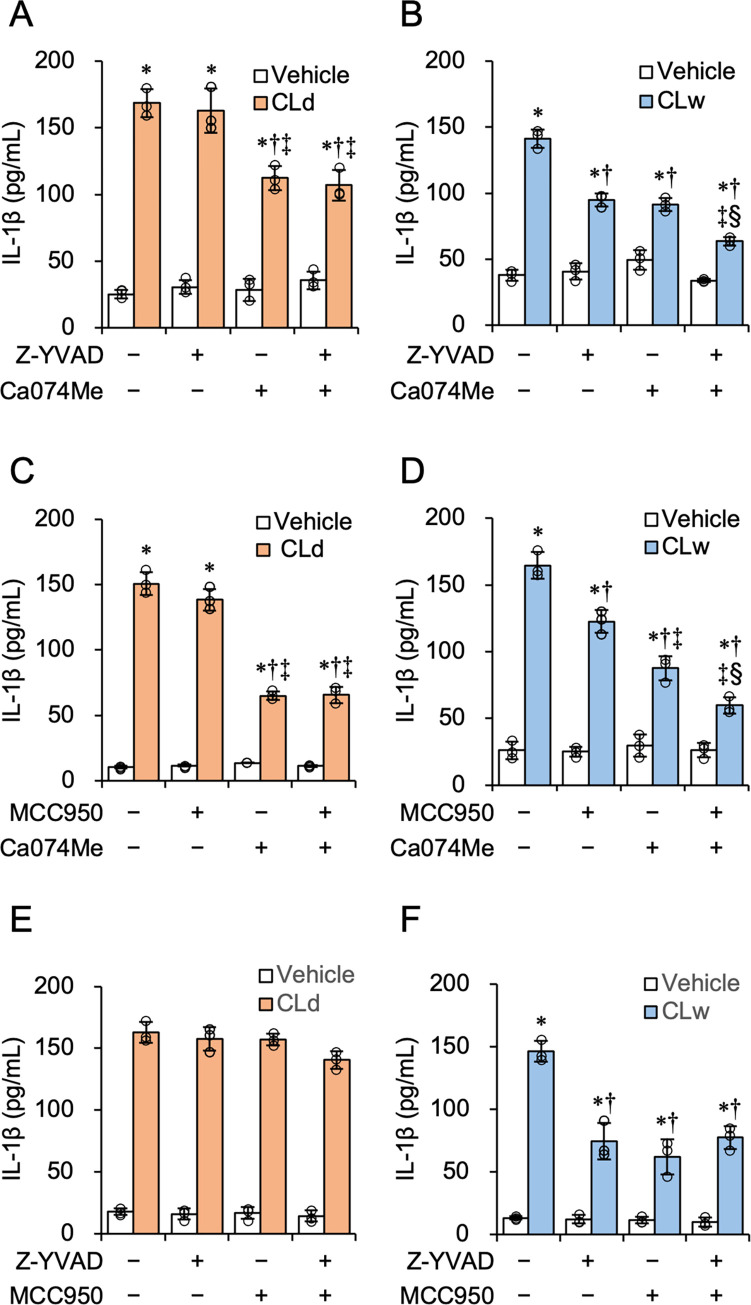
Influence of the simultaneous inhibition of the NLPR3 pathway and cathepsin B on the IL-1β-producing activity of CLd and CLw. LPS-primed THP-1 cells were pretreated with following inhibitors: 20 μM of the caspase-1 inhibitor Z-YVAD-fmk and/or 20 μM of the cathepsin B inhibitor Ca074Me (A and B); 2 μM of the NLRP3 inhibitor MCC950 and/or 20 μM Ca074Me (C and D); and 20 μM Z-YVAD-fmk and/or 2 μM MCC950 (E and F) or with vehicle for 1 h. Cells were then treated with 1 μM CLd (A, C, and E) or 10 μM CLw (B, D, and F) for 3 h. The vehicle controls for CLd and CLw are a medium containing 1% DMSO (A, C, and E) and a water-added medium (B, D, and F), respectively. TheIL-1β levels in the culture supernatants were assessed by ELISA. Data are presented as the mean ± SD (n = 3) and are representative of three independent experiments. *P < 0.05 compared with the vehicle (μc < μi); ^†^*P* < 0.05 compared with the no inhibitor control (μc > μi); ^‡^*P* < 0.05 compared with the single use of the first inhibitor (μc > μi); ^§^*P* < 0.05 compared with the single use of the second inhibitor (μc < μi) by one-way ANOVA followed by Dunnett’s test.

To identify the different mechanisms of IL-1β production by CLd and CLw, we used an inhibitor study. Inhibition of caspase-1 by Z-YVAD-fmk suppressed IL-1β production by CLw, but did not affect IL-1β production by CLd (Fig 5A). Similar results were obtained for the NLRP3 inhibitor MCC950 (Fig 5B) and potassium efflux inhibition by 25 mM KCl (Fig 5C). In contrast, inhibition of cathepsin B by Ca074Me reduced IL-1β production by CLd and CLw (Fig 5D). The inhibition of phagocytosis by cytochalasin D also reduced the production by CLd and CLw (Fig 5E). Furthermore, simultaneous inhibition of caspase-1 and cathepsin B revealed that IL-1β production by CLd was not significantly affected by caspase-1 inhibition (Fig 6A), while that by CLw was additively decreased (Fig 6B). Similar results were obtained for the simultaneous inhibition of NLRP3 and cathepsin B (Fig 6C and 6D). Simultaneous inhibition of caspase-1 and NLRP3 did not additively decrease IL-1β production by CLw (Fig 6F), which was different from the simultaneous inhibition of cathepsin B and caspase-1 or cathepsin B and NLRP3. Simultaneous inhibition of caspase-1 and NLRP3 did not affect CLd-induced IL-1β production (Fig 6E). These simultaneous inhibitions did not affect the cytotoxic activities of CLd and CLw (S3 Fig).

Thus, stimulation of IL-1β production by CLd was not associated with the NLRP3 inflammasome pathway, whereas CLd and CLw share a common IL-1β-inducing mechanism involving the lysosomal enzyme cathepsin B and the process of endocytosis. Furthermore, based on the results of the simultaneous inhibition study, the NLRP3 inflammasome pathway and the pathway regulated by cathepsin B may independently contribute to the induction of IL-1β production by CLw.

### Identification of the bioactive sources of CLd and CLw

**Fig 7:**
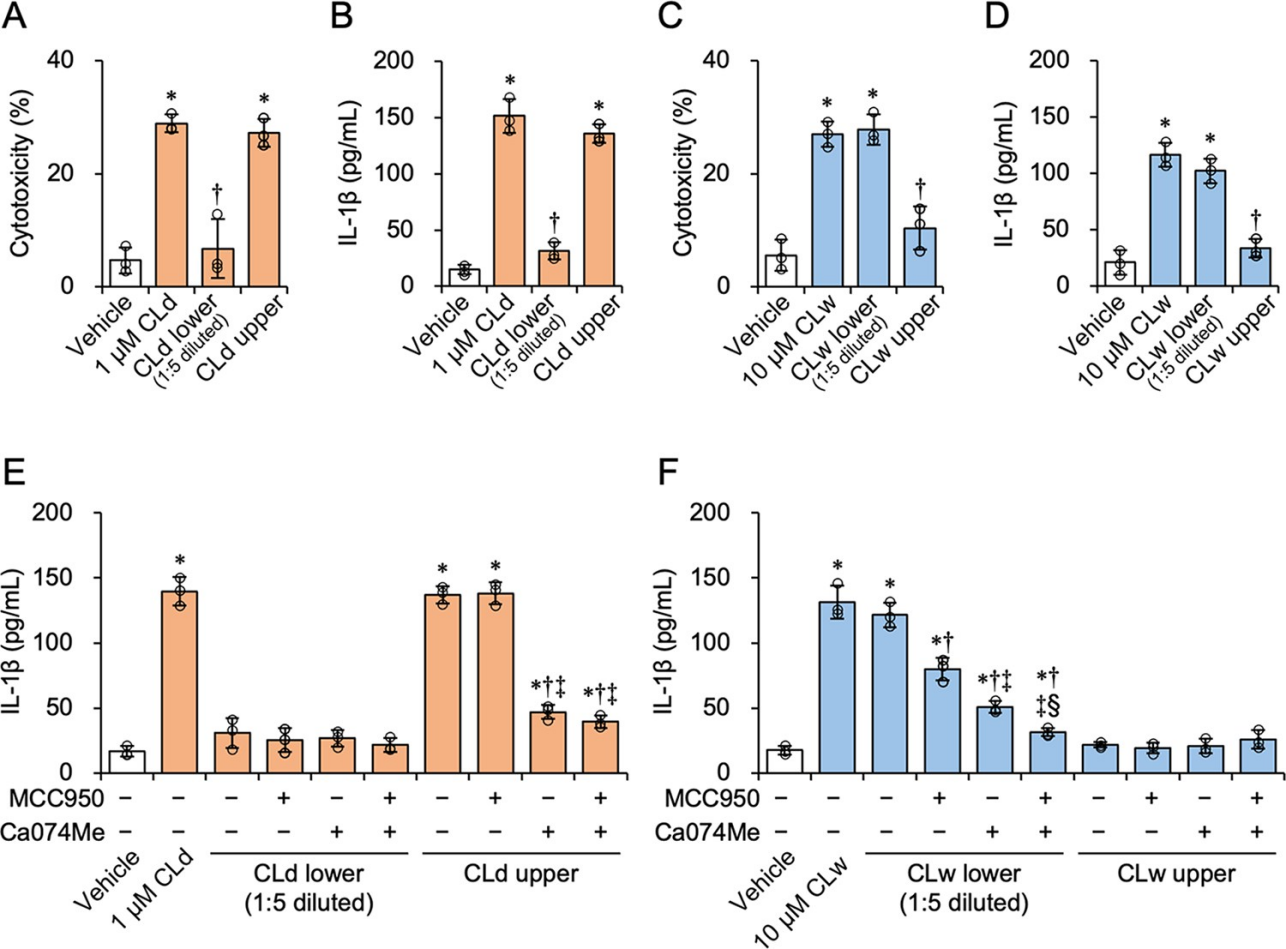
Identification of bioactive fractions of CLd and CLw. A-D: Fractionation of CLd and CLw was performed by centrifugation at 100 × *g* for 5 min at room temperature. The upper fractions were undiluted, and the lower fractions were diluted five times to restore to their original volume, being used equally with the uncentrifuged peptide solutions. The LPS-primed THP-1 macrophage-like cells were treated with the upper and lower fractions of CLd and CLw for 3 h. The vehicle controls for CLd and CLw are a medium containing 1% DMSO (A and B) and a water-added medium (C and D), respectively. Cytotoxicity was quantified by LDH release assay (A and C), and IL-1β levels in the culture supernatants were assessed by ELISA (B and D). Data are presented as the mean ± SD (n = 3) and are representative of three independent experiments. *P < 0.05 compared with the vehicle by one-way ANOVA followed by Dunnett’s test (μc < μi). E and F: For simultaneous inhibition assay, the LPS-primed THP-1 macrophage-like cells were pretreated with 2 μM of the NLRP3 inhibitor MCC950 and/or 20 μM Ca074Me or with vehicle for 1 h. Cells were then treated with the upper and lower fractions of CLd (E) and CLw (F) for 3 h. The vehicle controls for CLd and CLw are a medium containing 2% DMSO (E) and a medium containing 1% DMSO (F), respectively. The IL-1β levels in the culture supernatants were assessed by ELISA. Data are presented as the mean ± SD (n = 3) and are representative of three independent experiments. *P < 0.05 compared with the vehicle (μc < μi); ^†^*P* < 0.05 compared with the no inhibitor control (μc > μi); ^‡^*P* < 0.05 compared with the single use of the first inhibitor (μc > μi); ^§^*P* < 0.05 compared with the single use of the second inhibitor (μc < μi) by one-way ANOVA followed by Dunnett’s test.

Our results suggested that the bioactivities of CLd and CLw differ with respect to IL-1β production. As CLw contained many insoluble particles (Fig 1), we investigated whether the particles exerted the bioactivity of CLw. The stock solution of CLw was fractionated by centrifugation to separate it into an upper fraction and a lower fraction enriched in particles (S4 Fig). CLd was fractioned in the same manner, although CLd did not contain any visible particles. The upper fractions were undiluted, and the lower fractions were diluted five times to restore their original volume and used equally with uncentrifuged peptide solutions. For CLd, the cytotoxicity and IL-1β-producing activity of the upper fraction were similar to those of the uncentrifuged solution (1 μM CLd), and the activities were not increased in the lower fraction (Fig 7A and 7B). For CLw, the cytotoxicity and IL-1β-producing activity of the upper fraction were considerably reduced, and the activities were increased in the lower fraction (Fig 7C and 7D). The IL-1β-producing activities of the upper and lower fractions of CLd were reduced by cathepsin B inhibition, but not by NLRP3 inhibition (Fig 7E). This result was equivalent to that of the uncentrifuged CLd, shown in Fig 6C. The IL-1β-producing activity of the lower fraction of CLw was additively reduced by the NLRP3 and cathepsin B inhibitors (Fig 7F), consistent with the result observed with uncentrifuged CLw (Fig 6D). The cytotoxicity of the upper and lower fractions of CLd and CLw was not affected by the inhibitors (S5 Fig). These results indicate that the particle-enriched fraction of CLw is responsible for the bioactivity of CLw, and that the active component of CLw is different from that of CLd.

**Fig 8:**
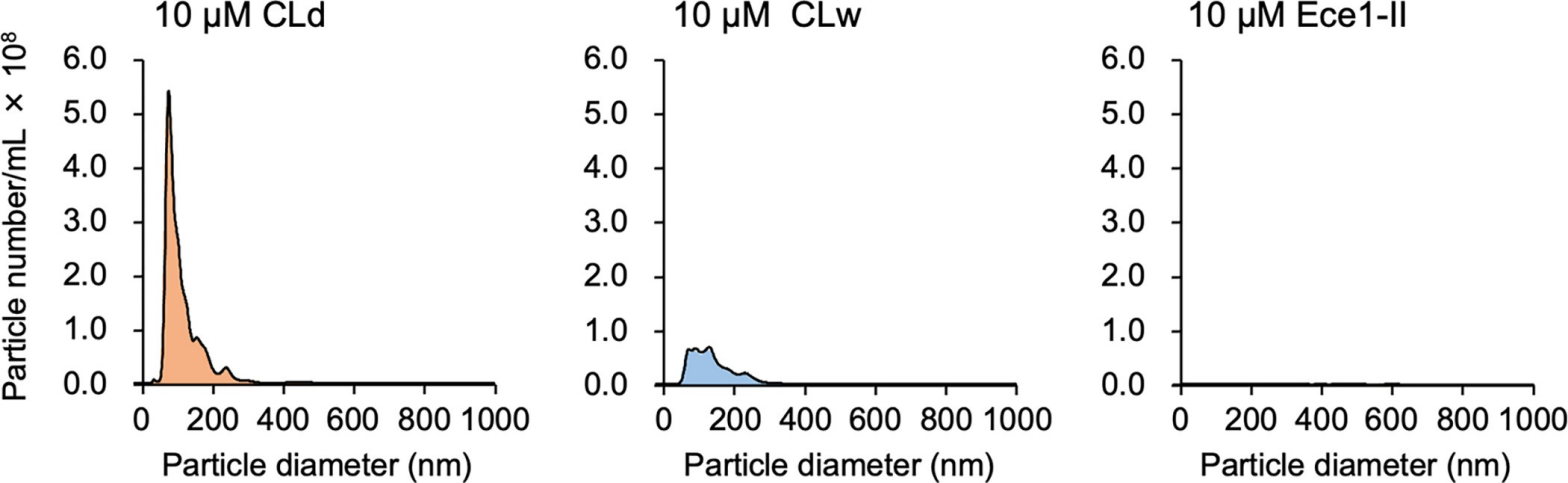
Detection of nanoparticles in CLd and CLw. Analyses of the number and size of nanoparticles in 10 μM CLd, CLw, and Ece1-II control peptide were performed using a NanoSight NS300 nanoparticle characterizer. Data are presented as the mean of five independent experiments.

Finally, we introduced nanoparticle tracking analysis to investigate the solubility of the synthetic candidalysin peptide in CLd. The results indicated that the peptide in CLd was present as nanoparticles with a size of 96 nm (Fig 8). Importantly, CLw had a much smaller amount of nanoparticles than CLd. This result suggests that solubilized candidalysin can be present as nanoparticles, probably owing to the bioactivity of candidalysin. In water, the synthetic candidalysin mostly aggregated to form microparticles and was only partly solubilized to form nanoparticles.

## Discussion

Candidalysin is a peptide toxin with hydrophobic properties. The synthetic candidalysin has been widely used in various experiments to investigate its bioactivity, although its solubility has not been well characterized. In most previous studies, the peptide was dissolved in water and used it at concentrations ≥10 μM [2, 5, 7, 18–20]. In this study, we showed that the water-dissolved peptide was not completely solubilized at a concentration of 10 μM, and the solution (CLw) contained abundant insoluble microparticles (≤1×10^5^/ml) and smaller amounts of soluble nanoparticles (≤8×10^7^/ml) than CLd (Figs 1 and 8). In contrast, the peptide was completely soluble in DMSO and exclusively present as nanoparticles (≤6×10^8^/ml), and the DMSO solution (CLd) exerted bioactivity even at concentrations ≤1 μM. Therefore, it is possible that certain results in previous studies using the water-dissolved synthetic peptide at concentrations ≥10 μM were produced by insoluble microparticles.

Although our results suggest that candidalysin tends to aggregate in water, this may be very different from *C*. *albicans*-produced candidalysin. In the case of the synthetic candidalysin, a large amount of the peptide (with a high hydrophobic property) may be present in a limited volume of water within a certain container that has a limited volume. Under such a condition, the peptide is highly clustered together and naturally becomes prone to aggregation. On the other hand, in the case of *C*. *albicans*-produced candidalysin, the amount of peptide produced from a hypha is considerably small; therefore, the amount of peptides present in a certain volume of water will not be so large. Additionally, since candidalysin is produced through the processing of Ece1 protein along with other peptides with various hydrophilicities, such an environment is unlikely to be one in which only a single hydrophobic peptide is clustered together. Therefore, *C*. *albicans*-produced candidalysin is unlikely to aggregate in water.

We demonstrated that CLd had stronger cytotoxicity and IL-1β-producing activity than CLw (Figs 2 and 4). This is consistent with a previous report preferred to use the DMSO solution because of the strength of its bioactivity in human oral epithelial cells [21]. Our data indicates that the water insolubility of the candidalysin peptide affects the strength of the activities. As shown above, CLw contains both insoluble microparticles and soluble nanoparticles, whereas CLd only contains nanoparticles, particularly those with a size of 96 nm (Figs 1 and 8). The insoluble microparticles exerted weak cytotoxic and NLRP3-dependent IL-1β-producing activity (Fig 7 and S5 Fig). In contrast, soluble nanoparticles exert considerably strong cytotoxic and cathepsin B-dependent IL-1β-producing activity. Thus, the intrinsic IL-1β-producing activity of candidalysin is presumably exerted under solubilized conditions as nanoparticles, whereas cytotoxic activity is exerted regardless of particle size.

The cytotoxicity of CLd and CLw is commonly exhibited through cell membrane damage and phagocytosis (actin polymerization), but not through activation of apoptotic caspases, the NLRP3 inflammasome pathway, or cathepsin B (Fig 3). Conversely, the IL-1β-producing activities of CLd and CLw are commonly mediated by phagocytosis as well as cathepsin B (Figs 5 and 6). The NLRP3 inflammasome pathway was involved in the IL-1β-producing activity of CLw, and fractionation of CLw revealed that this activity depends on insoluble microparticles (Fig 7). The cytotoxicity of CLw is induced through phagocytosis but not through the NLRP3 inflammasome pathway, while the IL-1β-producing activity of CLd (or nanoparticles) depends on cathepsin B but not on NLRP3 (Fig 5). These observations collectively suggest that NLRP3-dependent activity of the synthetic candidalysin peptide is caused by insoluble microparticles and is not an intrinsic activity of candidalysin.

When the clearance of phagocytosed components is inadequate, destabilization of phagolysosomes occasionally results in lysosomal rupture and release of cathepsin B, causing activation of NLRP3 [22, 23]. This NLRP3-dependent pathway can be activated by crystalline particles such as monosodium urate crystals, silica, and asbestos [24–26]. Therefore, it is possible that the induction of IL-1β production by insoluble microparticles in CLw is mediated by such a process, because its activity was reduced by inhibition of phagocytosis and cathepsin B, as well as inhibition of the NLRP3 inflammasome pathway (Figs 5E, 6B and 6D). Consistently, the activity of the water-dissolved candidalysin peptide can be inhibited by cytochalasin D and is mediated through the NLRP3 pathway [7]. Interestingly, the IL-1β-producing activity of CLd, which is independent from the NLRP3 pathway, was also reduced by the inhibition of phagocytosis and cathepsin B (Figs 5E, 6A and 6C). Although the reason is unclear, it is possible that CLd activates an alternative IL-1β processing machinery in a cathepsin B-dependent manner [27].

In summary, this study demonstrated that the bioactivity of synthetic candidalysin, especially the IL-1β-producing activity, is affected by solubility and by the solvent employed. The peptide dissolved in water is present mainly as insoluble microparticles with cytotoxic and NLRP3-dependent IL-1β-producing activity. Additionally, low amounts of soluble nanoparticles are present in the water solution, and these exhibit cytotoxicity and cathepsin B-dependent IL-1β-inducing activity. The peptide dissolved in DMSO was present mainly as nanoparticles, exhibiting strong cytotoxicity and cathepsin B-dependent IL-1β-producing activity. Thus, our findings may not be useful in understanding the function of candidalysin in actual settings of *Candida* infection but may help to better understand the properties and intrinsic biological activities of candidalysin, to reexamine how to use the synthetic candidalysin peptide, and to lead to the discovery of novel biological activities of candidalysin. Future research using the synthetic candidalysin peptide should consider these properties. The mechanism of candidalysin activation by the cathepsin B-dependent IL-1β processing machinery should be addressed in a future study.

## Supporting information

S1 Fig Intracellular Ca^2+^ levels in CLd- or CLw-treated THP-1 macrophage-like cells. (PDF)

S2 Fig Influence of LPS treatment on the cytotoxicity of CLd and CLw. (PDF)

S3 Fig Influence of the simultaneous inhibition of the NLPR3 pathway and cathepsin B on the cytotoxicity of CLd and CLw. (PDF)

S4 Fig Fractionation of CLw. (PDF)

S5 Fig Influence of the simultaneous inhibition of the NLPR3 pathway and cathepsin B on the cytotoxicity of the fractions of CLd and CLw. (PDF)

S1 Protocol Measurement of intracellular calcium levels. (PDF)

S1 Raw images Raw uncropped images of western blots shown in Fig 4C. (PDF)

## References

[1] König A, Hube B, Kasper L. The Dual Function of the Fungal Toxin Candidalysin during Candida albicans—Macrophage Interaction and Virulence. Toxins (Basel). 2020;12: 469. doi: 10.3390/toxins12080469 32722029 PMC7471981

[2] Moyes DL, Wilson D, Richardson JP, Mogavero S, Tang SX, Wernecke J, et al. Candidalysin is a fungal peptide toxin critical for mucosal infection. Nature. 2016;532: 64–68. doi: 10.1038/nature17625 27027296 PMC4851236

[3] Richardson JP, Brown R, Kichik N, Lee S, Priest E, Mogavero S, et al. Candidalysins Are a New Family of Cytolytic Fungal Peptide Toxins. Hogan DA, editor. MBio. 2022. doi: 10.1128/mbio.03510-21 35073742 PMC8787473

[4] Brown R, Priest E, Naglik JR, Richardson JP. Fungal Toxins and Host Immune Responses. Front Microbiol. 2021;12: 1–19. doi: 10.3389/fmicb.2021.643639 33927703 PMC8076518

[5] Westman J, Plumb J, Licht A, Yang M, Allert S, Naglik JR, et al. Calcium-dependent ESCRT recruitment and lysosome exocytosis maintain epithelial integrity during Candida albicans invasion. Cell Rep. 2022;38: 110187. doi: 10.1016/j.celrep.2021.110187 34986345 PMC8755444

[6] Blagojevic M, Camilli G, Maxson M, Hube B, Moyes DL, Richardson JP, et al. Candidalysin triggers epithelial cellular stresses that induce necrotic death. Cell Microbiol. 2021; e13371. doi: 10.1111/cmi.13371 34085369 PMC8460601

[7] Kasper L, König A, Koenig P-A, Gresnigt MS, Westman J, Drummond RA, et al. The fungal peptide toxin Candidalysin activates the NLRP3 inflammasome and causes cytolysis in mononuclear phagocytes. Nat Commun. 2018;9: 4260. doi: 10.1038/s41467-018-06607-1 30323213 PMC6189146

[8] Franchi L, Muñoz-planillo R, Núñez G. Sensing and Reacting to Microbes via the Inflammasomes. Nat Immunol. 2012;13: 325–332. doi: 10.1038/ni.2231.Sensing 22430785 PMC3449002

[9] Paik S, Kim JK, Silwal P, Sasakawa C, Jo EK. An update on the regulatory mechanisms of NLRP3 inflammasome activation. Cell Mol Immunol. 2021;18: 1141–1160. doi: 10.1038/s41423-021-00670-3 33850310 PMC8093260

[10] Groslambert M, Py BF. Spotlight on the NLRP3 inflammasome pathway. J Inflamm Res. 2018;11: 359–374. doi: 10.2147/JIR.S141220 30288079 PMC6161739

[11] Kataoka H, Saeki A, Hasebe A, Shibata K-I, Into T. Naringenin suppresses Toll-like receptor 2-mediated inflammatory responses through inhibition of receptor clustering on lipid rafts. Food Sci Nutr. 2021;9: 963–972. doi: 10.1002/fsn3.2063 33598179 PMC7866581

[12] Mori T, Kataoka H, Into T. Effect of Myd88 deficiency on gene expression profiling in salivary glands of female non-obese diabetic (NOD) mice. J oral Biosci. 2021;63: 192–198. doi: 10.1016/j.job.2021.04.003 33933610

[13] Into T, Niida S, Shibata K. MyD88 signaling causes autoimmune sialadenitis through formation of high endothelial venules and upregulation of LTβ receptor-mediated signaling. Sci Rep. 2018;8: 14272. doi: 10.1038/s41598-018-32690-x 30250175 PMC6155371

[14] Rogiers O, Frising UC, Kucharíková S, Jabra-Rizk MA, van Loo G, Van Dijck P, et al. Candidalysin crucially contributes to nlrp3 inflammasome activation by Candida albicans hyphae. MBio. 2019;10. doi: 10.1128/mBio.02221-18 30622184 PMC6325245

[15] Ho J, Yang X, Nikou SA, Kichik N, Donkin A, Ponde NO, et al. Candidalysin activates innate epithelial immune responses via epidermal growth factor receptor. Nat Commun. 2019;10. doi: 10.1038/s41467-019-09915-2 31127085 PMC6534540

[16] Netea MG, Simon A, van de Veerdonk F, Kullberg B-J, Van der Meer JWM, Joosten LAB. IL-1beta processing in host defense: beyond the inflammasomes. PLoS Pathog. 2010;6: e1000661. doi: 10.1371/journal.ppat.1000661 20195505 PMC2829053

[17] Afonina IS, Müller C, Martin SJ, Beyaert R. Proteolytic Processing of Interleukin-1 Family Cytokines: Variations on a Common Theme. Immunity. 2015;42: 991–1004. doi: 10.1016/j.immuni.2015.06.003 26084020

[18] Wu Y, Zeng Z, Guo Y, Song L, Weatherhead JE, Huang X, et al. Candida albicans elicits protective allergic responses via platelet mediated T helper 2 and T helper 17 cell polarization. Immunity. 2021; 1–16. doi: 10.1016/j.immuni.2021.08.009 34506733 PMC8585696

[19] Ho J, Wickramasinghe DN, Nikou SA, Hube B, Richardson JP, Naglik JR. Candidalysin Is a Potent Trigger of Alarmin and Antimicrobial Peptide Release in Epithelial Cells. Cells. 2020;9: 14–20. doi: 10.3390/cells9030699 32178483 PMC7140650

[20] Swidergall M, Solis N V., Millet N, Huang MY, Lin J, Phan QT, et al. Activation of EphA2-EGFR signaling in oral epithelial cells by Candida albicans virulence factors. PLoS Pathogens. 2021. doi: 10.1371/journal.ppat.1009221 33471869 PMC7850503

[21] Hanaoka M, Domae E. IL-1α released from oral epithelial cells upon candidalysin exposure initiates an early innate epithelial response. Int Immunol. 2021;33: 161–170. doi: 10.1093/intimm/dxaa070 33038250

[22] Campden RI, Zhang Y. The role of lysosomal cysteine cathepsins in NLRP3 inflammasome activation. Arch Biochem Biophys. 2019;670: 32–42. doi: 10.1016/j.abb.2019.02.015 30807742

[23] Chevriaux A, Pilot T, Derangère V, Simonin H, Martine P, Chalmin F, et al. Cathepsin B Is Required for NLRP3 Inflammasome Activation in Macrophages, Through NLRP3 Interaction. Front cell Dev Biol. 2020;8: 167. doi: 10.3389/fcell.2020.00167 32328491 PMC7162607

[24] Martinon F, Pétrilli V, Mayor A, Tardivel A, Tschopp J. Gout-associated uric acid crystals activate the NALP3 inflammasome. Nature. 2006;440: 237–241. doi: 10.1038/nature04516 16407889

[25] Hornung V, Bauernfeind F, Halle A, Samstad EO, Kono H, Rock KL, et al. Silica crystals and aluminum salts activate the NALP3 inflammasome through phagosomal destabilization. Nat Immunol. 2008;9: 847–856. doi: 10.1038/ni.1631 18604214 PMC2834784

[26] Dostert C, Pétrilli V, Van Bruggen R, Steele C, Mossman BT, Tschopp J. Innate immune activation through Nalp3 inflammasome sensing of asbestos and silica. Science. 2008;320: 674–7. doi: 10.1126/science.1156995 18403674 PMC2396588

[27] Dinarello CA. Interleukin-1 in the pathogenesis and treatment of inflammatory diseases. Blood. 2011;117: 3720–32. doi: 10.1182/blood-2010-07-273417 21304099 PMC3083294

