# Supplementary material for: Solubility affects IL-1β-producing activity of the synthetic candidalysin peptide
Source: PLoS One. 2022 Aug 30;17(8):e0273663. doi: 10.1371/journal.pone.0273663 (PMC9426886; doi:10.1371/journal.pone.0273663)
Supplement: S1 Fig — (PDF) [file pone.0273663.s001.pdf]

S1 Fig

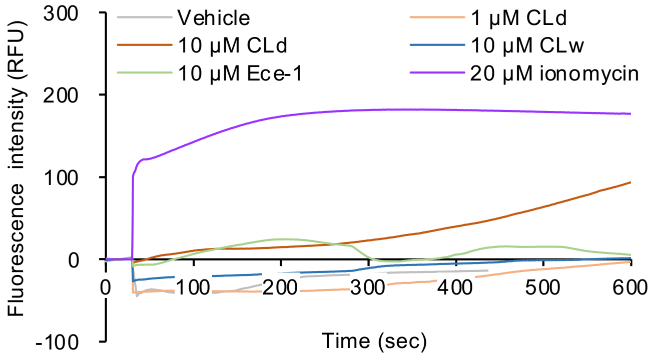

S1 Fig. Intracellular Ca^{2+} levels in CLd- or CLw-treated THP-1 macrophage-like cells. Differentiated THP-1 macrophage-like cells were preloaded with Fluo-4 AM followed by stimulation with the indicated stimulators. The fluorescent intensity of Fluo 4 was monitored for 600 s. Each color line indicates each stimulator: gray line, vehicle control (1% DMSO); orange line, 1 μM CLd; red line, 10 μM CLd; blue line, 10 μM CLw; yellow green line, 10 μM Ece-1; and purple line, 20 μM ionomycin. Representative results of at least three independent experiments are shown.
