# Supplementary material for: Solubility affects IL-1β-producing activity of the synthetic candidalysin peptide
Source: PLoS One. 2022 Aug 30;17(8):e0273663. doi: 10.1371/journal.pone.0273663 (PMC9426886; doi:10.1371/journal.pone.0273663)
Supplement: S2 Fig — (PDF) [file pone.0273663.s002.pdf]

# S2 Fig

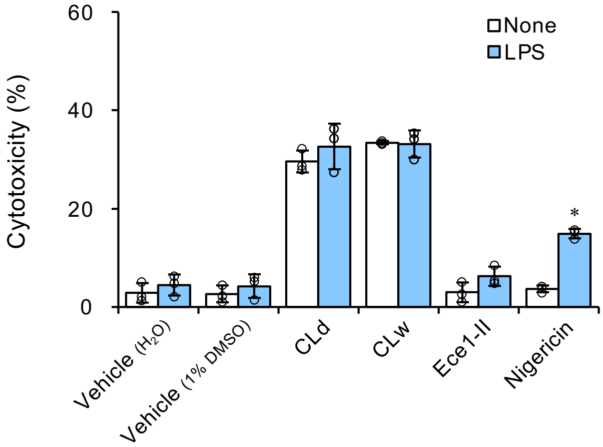


**S2 Fig. Influence of LPS treatment on the cytotoxicity of CLd and CLw.** Differentiated THP-1 cells were pretreated with 100 ng/mL LPS or vehicle for 4 h. Next, the cells were treated with 1 μM CLd or 10 μM CLw for 3 h. The vehicle controls for CLd and CLw are a medium containing 1% DMSO and a water-added medium ($H_2O$), respectively. Cytotoxicity was determined using an LDH release assay. Data are presented as mean ± SD (n=3) of three independent experiments. $^{*}P < 0.05$ compared with the none by one-way ANOVA followed by Dunnett's test ($\mu c < \mu i$).
