# Supplementary material for: Solubility affects IL-1β-producing activity of the synthetic candidalysin peptide
Source: PLoS One. 2022 Aug 30;17(8):e0273663. doi: 10.1371/journal.pone.0273663 (PMC9426886; doi:10.1371/journal.pone.0273663)
Supplement: S3 Fig — (PDF) [file pone.0273663.s003.pdf]

S3 Fig

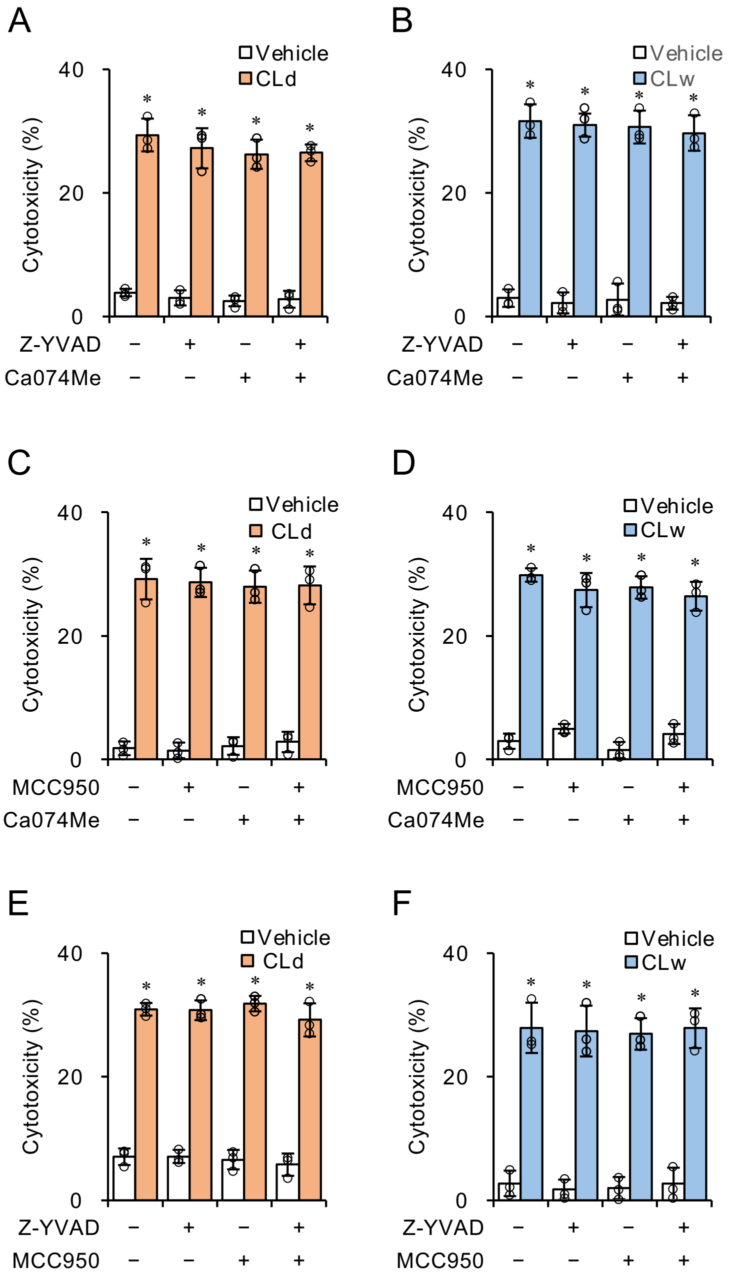

S3 Fig. Influence of the simultaneous inhibition of the NLPR3 pathway and cathepsin B on the cytotoxicity of CLd and CLw. Differentiated THP-1 cells were pretreated with the following inhibitors: 20 μM of the caspase-1 inhibitor Z-YVAD-fmk and/or 20 μM of the cathepsin B inhibitor Ca074Me (A and B), 2 μM of the NLRP3 inhibitor MCC950 and/or 20 μM Ca074Me (C and D), and 20 μM Z-YVAD-fmk and/or 2 μM MCC950 (E and F) or with vehicle for 1 h. Cells were then treated with 1 μM CLd (A, C, and E) or 10 μM CLw (B, D, and F) for 3 h. The vehicle controls for CLd and CLw are a medium containing 2% DMSO (A, C, and E) and a water-added medium (B, D, and F), respectively. Cytotoxicity was determined using an LDH release assay. Data are presented as mean ± SD (n=3) of three independent experiments. **P* < 0.05 compared with the vehicle by one-way ANOVA followed by Dunnett's test ($\mu_c < \mu_i$).
