# Supplementary material for: Solubility affects IL-1β-producing activity of the synthetic candidalysin peptide
Source: PLoS One. 2022 Aug 30;17(8):e0273663. doi: 10.1371/journal.pone.0273663 (PMC9426886; doi:10.1371/journal.pone.0273663)
Supplement: S4 Fig — (PDF) [file pone.0273663.s004.pdf]

S4 Fig

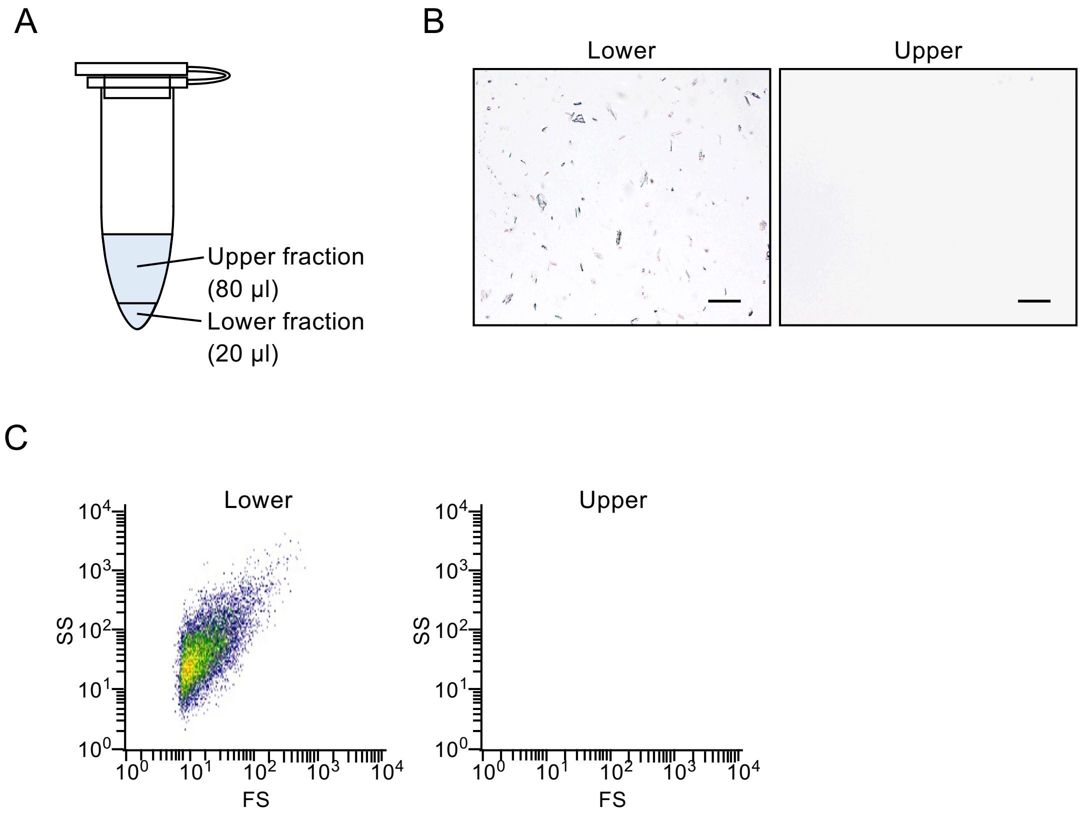

S4 Fig. Fractionation of CLw. A: Fractionation of a stock solution of CLw was performed by centrifugation at 100 × *g* for 5 min at room temperature. The upper fraction (80 μl) and the residual lower fraction (20 μl) were separated by discreet pipetting. B: Microscopic images of the upper and lower fractions of CLw. Images were captured at a magnification of 200 ×. Scale bar, 100 μm. C: Plots of the particles in the upper and lower fractions by flow cytometric FS and SS analysis. Representative results of at least three independent experiments are shown.
