# Supplementary material for: Solubility affects IL-1β-producing activity of the synthetic candidalysin peptide
Source: PLoS One. 2022 Aug 30;17(8):e0273663. doi: 10.1371/journal.pone.0273663 (PMC9426886; doi:10.1371/journal.pone.0273663)
Supplement: S5 Fig — (PDF) [file pone.0273663.s005.pdf]

S5 Fig

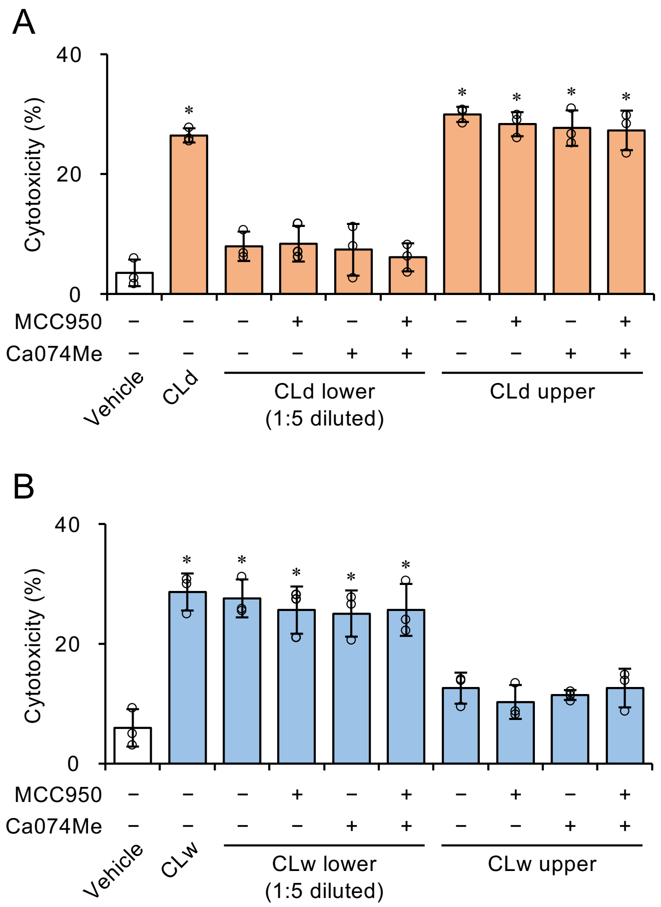

S5 Fig. Influence of the simultaneous inhibition of the NLPR3 pathway and cathepsin B on the cytotoxicity of the fractions of CLd and CLw. LPS-primed THP-1 cells were pretreated with 2 μM of the NLRP3 inhibitor MCC950 and/or 20 μM Ca074Me or vehicle for 1 h. Cells were then treated with the upper or lower fractions of CLd (A) and CLw (B) for 3 h. The vehicle controls for CLd and CLw are a medium containing 2% DMSO (A) and a water-added medium (B), respectively. Cytotoxicity was quantified using an LDH release assay. Data are presented as mean ± SD (n=3) of three independent experiments. *$P < 0.05$ compared with the vehicle by one-way ANOVA followed by Dunnett's test ($\mu c < \mu i$).
