# Supplementary material for: Solubility affects IL-1β-producing activity of the synthetic candidalysin peptide
Source: PLoS One. 2022 Aug 30;17(8):e0273663. doi: 10.1371/journal.pone.0273663 (PMC9426886; doi:10.1371/journal.pone.0273663)
Supplement: S1 Protocol — (PDF) [file pone.0273663.s006.pdf]

S1 Protocol

Measurement of intracellular calcium levels.

Intracellular calcium levels were measured using the Calcium Kit-Fluo 4 kit (Dojindo), following the manufacturer's instructions. Briefly, differentiated THP-1 cells were plated at 1×10^5 cells/well in 96-well black microplates (Nunc). Cells were pretreated with a loading medium containing Fluo-4 AM and incubated for 1 h. After the medium was changed to recording medium and washed once with phosphate buffered saline (PBS), the cells were stimulated with each compound. The fluorescence of Fluo-4 was detected using the GloMax Discover GM3000 microplate reader (Promega). Fluorescence intensity was measured at 1 s intervals for 600 s.
