# Supplementary material for: Solubility affects IL-1β-producing activity of the synthetic candidalysin peptide
Source: PLoS One. 2022 Aug 30;17(8):e0273663. doi: 10.1371/journal.pone.0273663 (PMC9426886; doi:10.1371/journal.pone.0273663)
Supplement: S1 Raw images — (PDF) [file pone.0273663.s007.pdf]

S1 Raw Images

Cleaved IL-1β (p17)

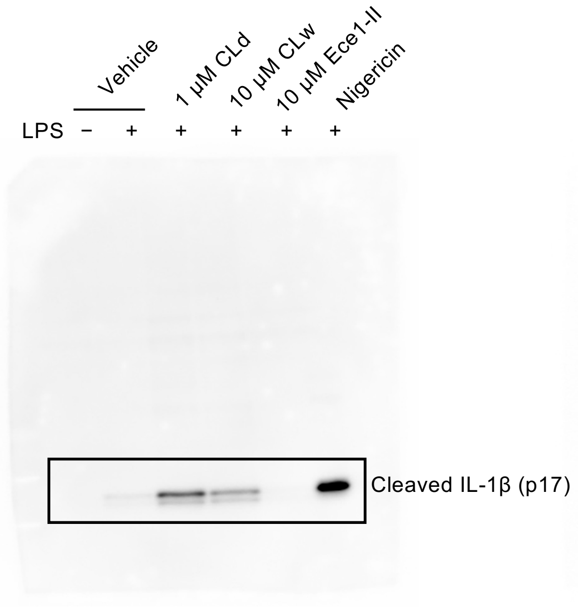

Pro IL-1β (Lysate)

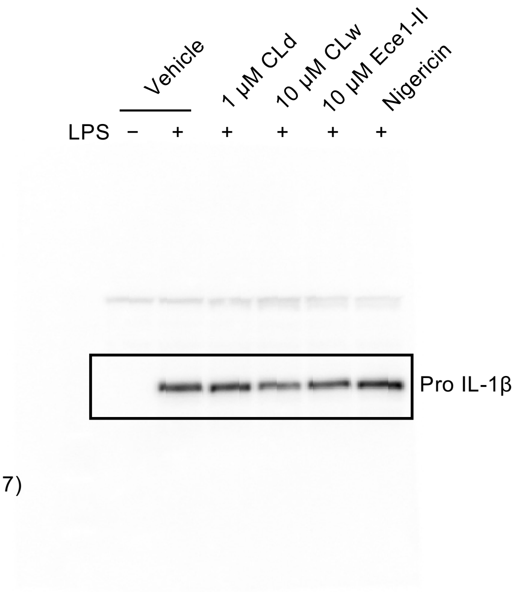

Tubulin (Lysate)

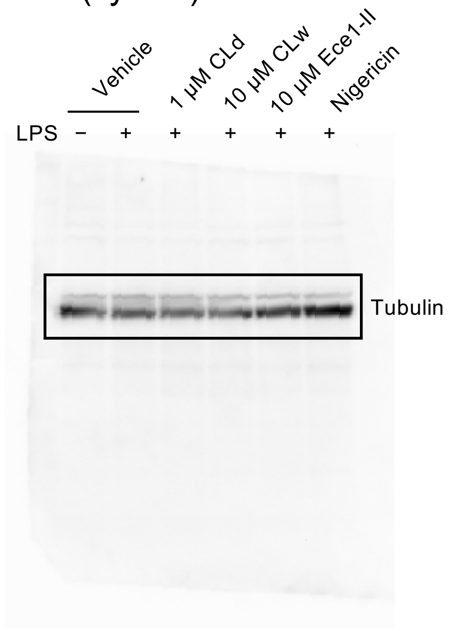

S1 Raw images. Raw uncropped images of western blots shown in Fig 4C.
